# Counting Enhances Kindergarteners’ Mappings of Number Words Onto Numerosities

**DOI:** 10.3389/fpsyg.2020.00153

**Published:** 2020-02-13

**Authors:** Winnie Wai Lan Chan

**Affiliations:** Department of Psychology, The University of Hong Kong, Pokfulam, Hong Kong

**Keywords:** counting, mapping, kindergarteners, numbers, story

## Abstract

We can make sense of how many a number represents by mapping the symbolic number word system onto the non-symbolic, approximate number system. This study adopted an intervention design to examine whether counting is essential in driving the formation of such symbolic-non-symbolic mappings. We compared kindergarteners’ mapping ability after reading stories (1) without cardinal labels, or (2) with cardinal labels, or (3) with cardinal labels and verbal counting. Results showed that children who had counted when reading the stories showed better mapping between number words and their approximate representations than their peers in the other two conditions – suggesting that counting plays a role in developing early symbolic-non-symbolic mappings. Such findings provides empirical support for parents and early educators to enhance young children’s mappings between numbers words and approximate representations through counting activities – such as counting the items in the story books while reading to children.

## Introduction

Well before receiving formal education, children have already started to make sense of the magnitudes represented by number words. To develop such sense, children need to map number words onto their preexisting, intuitive sense of numerosities. How well children can perform this mapping significantly differentiates their mathematical achievement (Mundy and Gilmore, 2009). In this study, we examined whether counting could facilitate young children’s mappings of number words onto numerosities.

Our intuitive sense of numerosities comes from the early non-symbolic representation of quantities known as the approximate number system (Xu and Spelke, 2000), which enables us to compare, add, and subtract large quantities without using words or digits (McCrink and Wynn, 2004; Barth et al., 2005; Wood and Spelke, 2005). Previous studies of human infants (Xu and Spelke, 2000; Lipton and Spelke, 2003), adults (Van Oeffelen and Vos, 1982; Barth et al., 2003), and animals (McComb et al., 1994) have found evidence for such a system. Accuracy when discriminating two sets of numerosities depends on their ratio. This ratio dependency, known as Weber’s Law, is a key characteristic of the approximate number system. The closer the two quantities are (i.e., the nearer their ratio to 1), the harder the discrimination is. The Weber ratio limit improves throughout development (Halberda and Feigenson, 2008): While 6-month-old infants can distinguish quantities at a 1:2 ratio (e.g., 14 vs. 28, but not 14 vs. 21), 9-month-old infants can do so at a 2:3 ratio (e.g., 14 vs. 21, but not 14 vs. 16) (Lipton and Spelke, 2003) and adults can do so at a 7:8 ratio (e.g., 14 vs. 16) (Van Oeffelen and Vos, 1982).

In addition to the preexisting approximate number system, we acquire the symbolic number system, which is language-dependent and thus unique to humans. Symbolic representations enable us to manipulate exact quantities. People performing exact arithmetic show greater activation of secondary language areas in the brain than those performing approximate arithmetic (Dehaene et al., 1999). While exact arithmetic skills acquired using one language are difficult to transfer to another language, approximate arithmetic skills are independent of the language in use (Dehaene et al., 1999).

The symbolic and non-symbolic systems are mapped onto each other. When comparing two digits, both children and adults are slower and less accurate when the digits are numerically closer than when they are farther part (e.g., 3 vs. 4 as opposed to 3 vs. 8) (Moyer and Landauer, 1967; Dehaene et al., 1990; Temple and Posner, 1998). This is known as the numerical distance effect, which mirrors the Weber ratio limit of non-symbolic representations and is taken as evidence for the interference of the preexisting, non-symbolic system on the symbolic system due to the mapping between the two. The size of the numerical distance effect is taken as an indirect measure of the precision of the symbolic–non-symbolic mapping (Rousselle and Noël, 2007; Holloway and Ansari, 2008). Mundy and Gilmore (2009) used a mapping task to assess children’s mapping ability directly. Children were asked to match a target number with one of the two arrays of dots provided or vice versa. Results show that children can map between symbolic and non-symbolic representations of numbers in both directions and that this ability develops at around age 6 to 8. Gilmore et al. (2007) have also found that young children can build on their non-symbolic number system to perform symbolic arithmetic. Mapping between symbolic and non-symbolic systems is also supported by neuropsychological findings, which show that impairments in either system are associated with impairments in the other (Dehaene et al., 1998).

Because the symbolic and non-symbolic systems are mapped onto each other, the skills derived from these two systems appear to be somewhat related. Indeed, Mazzocco et al. (2011a) have shown that preschoolers’ acuity of the approximate number system predicts their later performance in school mathematics. However, Inglis et al. (2011) have pointed out that such association changes with age and that the approximate number representations may not be the key factor accounting for the gaps in mathematical performance among adults. Previous studies focusing on children have found that mathematical learning difficulty is associated with poor acuity of the approximate number system (Mazzocco et al., 2011b) and that brief non-symbolic, approximate number practice enhances subsequent exact symbolic arithmetic (Hyde et al., 2014). Note that some studies have found no association between children’s acuity of the approximate number system and their symbolic number skills (e.g., Sasanguie et al., 2014). Such inconsistency may be due to the variation of tasks being used across different studies. For example, different measures of symbolic tasks were used by Hyde et al. (2014); symbolic arithmetic) and Sasanguie et al. (2014); digit comparison).

Some studies have suggested that children’s mapping ability has to do with their verbal counting ability. Sarnecka et al. (2015) have shown that children start to develop a mental representation of large numbers after learning to count. Lipton and Spelke (2005) have found that children can map number words onto numerosities within their counting range but not beyond. In particular, those who can count to 60, can provide verbal estimates that increase linearly with numerosity for sets ranging from 20 to 60, whereas those who can count to 100 can do so for sets ranging from 20 to 120. This leads the authors to conclude that number words are mapped onto the approximate representations as soon as the children learn to count to those words. Similarly, Barth et al. (2009) have shown that children who can count to 35 cannot produce verbal estimates increasing with numerosity for sets ranging from 60 to 140, whereas those who can count beyond 60 are able to do so. Surprisingly, they have found that children who cannot count to 35 can provide verbal estimates increasing with numerosity for large sets. Hence, it seems somewhat possible that children can develop partial mapping ability outside their counting range. This raises the question of whether counting is required for the development of mapping ability.

It remains unclear whether the development of symbolic–non-symbolic mapping ability depends on counting. All relevant studies so far have been correlational and thus unable to indicate any causal relationship. To fill this gap, we have used intervention design to examine whether children’s mapping ability depends on their counting skill. Here we asked an important question: Can practice of counting enhance children’s mappings of number words onto approximate representations? To address the question, we compared children’s mapping ability after attending story reading sessions in which (1) the narrator did not label the cardinality of items in the story (i.e., control condition), (2) the narrator labeled the cardinality of the items in the story (i.e., label condition), or (3) the narrator labeled the cardinality of the items and then counted them with the children (i.e., label-and-count condition). If the counting is required in developing symbolic–non-symbolic mappings, children having counted the items in the story with the narrator (i.e., label-and-count condition) would show better mappings. If, however, hearing the cardinal labels of different sets is sufficient to help children map between the symbolic and non-symbolic systems, children in both label and label-and-count conditions would show equally better mappings.

## Materials and Methods

### Participants

Eighty-nine Chinese kindergarteners (mean age = 4.5 years; 40 boys and 49 girls) were recruited from a kindergarten in Hong Kong. All the children participated in the Pre-primary Education Voucher Scheme and their tuition fees were fully subsidized by the government. Based on a power analysis (G^∗^Power Version 3.1.9.2) (Faul et al., 2007), we determined that a sample of 72 children would achieve 85% power to detect an effect size of 0.40 with an alpha of 0.05. All children participated with written parental consent. Ethical approval had been obtained from the Human Research Ethics Committee of the author’s University prior to data collection.

### Materials and Procedure

#### Story Reading Sessions

##### Materials

**FIGURE 1:**
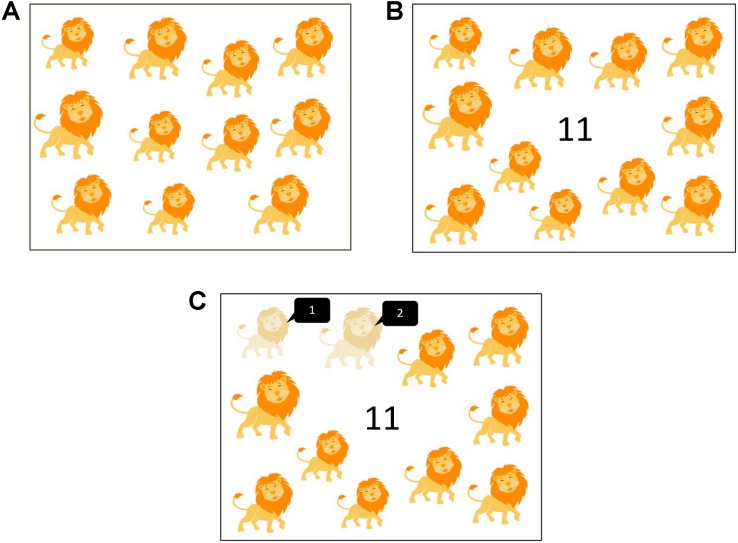
The pictures used in different conditions. In **(A)** control condition, only pictures were shown. In **(B)** label condition, an Arabic number representing the quantity of items appeared at the center. In **(C)** label-and-count condition, the narrator clicked on the mouse to lighten each item while he counted with the children. The black conversation boxes were not shown in the actual slides.

The story reading materials were developed based on ten children’s stories. Two versions of PowerPoint slides were constructed for each story. Each version contained 12 to 13 slides. Each slide showed pictures of one set of items (e.g., lions, mice, and trees) from the story without any printed words. The items were chosen to match with the content of the story. The quantity of items on each slide fell into one of the five ranges of set sizes: 1 to 10, 11 to 20, 21 to 30, 31 to 40, and 41 to 50. Each range of set size was adopted at least twice in each version of a story. Table 1 shows the set sizes of items included in each story. On each slide, the items were randomly positioned, and their physical size varied. For each story, the two versions of slides showed the same type of items (e.g., both versions showing lions on the first slide, mice on the second slide, and so on) but in different set sizes (e.g., on the first slide, one version showing 5 lions and the other showing 9 lions) (see Figure 1). The stories were presented in three reading conditions: label, label-and-count, and control. An Arabic number representing the set size appeared in the middle of each slide in both the label and label-and-count conditions, but not in the control condition. On each slide in the label-and-count condition, one item would be lightened (75% transparent) upon a mouse click by the experimenter. To keep track of the items during one-on-one counting, continual clicking on the mouse would lighten the items one by one.

**TABLE 1:** The exact set sizes of items included in each story.

		**Ranges of set sizes**				
	**Version**	**1 to 10**	**11 to 20**	**21 to 30**	**31 to 40**	**41 to 50**
Story 1	1	1, 3, 4	11, 14, 15	23, 24	34, 33	41, 45
	2	2, 3, 5	13, 18, 20	26, 29	31, 35	46, 48
Story 2	1	2, 5	13, 18	22, 25, 26	36, 38, 39	43, 48, 50
	2	4, 9	16, 19	22, 23, 27	32, 34, 38	41, 44, 45
Story 3	1	1, 2, 3	12, 15, 17	21, 24	31, 35	48, 49
	2	2, 3, 9	12, 16, 19	22, 24	38, 39	45, 46
Story 4	1	4, 5	13, 18	25, 27, 29	32, 33, 37	41, 43, 46
	2	1, 8	13, 14	23, 27, 30	33, 34, 37	43, 49, 50
Story 5	1	3, 4, 10	13, 15, 18	24, 30	33, 36	44, 45
	2	2, 5, 6	12, 14, 16	21, 27	35, 38	42, 43
Story 6	1	8, 9	12, 14	23, 25, 27	32, 38, 40	42, 43, 46
	2	3, 9	11, 20	22, 28, 29	31, 33, 39	41, 45, 47
Story 7	1	1, 4, 6	12, 14, 16	23, 24	34, 35	49, 50
	2	2, 7, 9	14, 15, 19	21, 28	32, 33	46, 48
Story 8	1	7, 8	15, 19	26, 27, 28	33, 39, 40	41, 45, 46
	2	3, 8	12, 13	22, 23, 25	34, 37, 38	43, 45, 47
Story 9	1	2, 5, 6	13, 16, 19	26, 28	34, 35	42, 46
	2	1, 3, 10	12, 14, 16	22, 27	34, 37	48, 50
Story 10	1	4, 9	17, 18	24, 25, 29	33, 37, 40	44, 47, 50
	2	2, 4	13, 17	25, 26, 28	33, 36, 38	41, 42, 44

*The set sizes were presented in a fixed random order in each story.*

##### Procedures

All children attended a 15-minute story reading session each day for 2 weeks at school. All the sessions were conducted in Chinese by the same experimenter. Altogether there were ten sessions (five sessions per week). They were conducted in a group. In each session, the experimenter read aloud two stories. Ten different stories were told in the first week and repeated in another version with the same storyline but different quantities of items on each slide in the second week. The experimenter showed the PowerPoint slides of the story on a projector screen while he was telling the story. The children were randomly assigned to one of the three reading conditions: the control group (*n* = 29; mean age = 4.6 years; 13 boys), label group (*n* = 30; mean age = 4.5 years; 14 boys), or label-and-count group (*n* = 30; mean age = 4.5 years; 13 boys). There was no significant difference in age across the three groups of children [*F*(2,85) = 0.31, *p* = 0.74]. In both the label and label-and-count conditions, the experimenter specified the quantity of items on each slide (e.g., “Once upon a time there lived three lions.”), whereas in the control condition, he used “some” to replace all the quantity words (e.g., “Once upon a time there lived some lions.”). In the label-and-count condition, the experimenter asked the children to count the items on each slide with him (e.g., “Once upon a time there lived three lions. Let’s count together: one, two, three; three lions.”). For each count, he clicked on the mouse to lighten each item until all items were counted. The experimenter did not count in the other two conditions. Note that the experimenter in the control and label conditions read more slowly (e.g., longer and more frequent pauses between sentences and between slides) and stayed on each slide for a longer time (e.g., asking the children to look carefully at the pictures on each slide) than he did in the label-and-count condition to ensure comparable duration per session across the three conditions. Based on our observation, the children in this condition did not show any sign of losing interest nor counting the items themselves during the presentation.

#### Symbolic and Non-symbolic Assessment

All children completed a battery of symbolic and non-symbolic tasks twice, before the first reading session (pretest) and after the last reading session (posttest). All the tasks were run on a computer. Note that the tasks had been trialed out among kindergartners in a pilot study before actual data collection to ensure that they were age-appropriate for young children.

##### Numerosity comparison

This task was used to assess the acuity of the non-symbolic, approximate number system. We used the Panamath program (Halberda et al., 2008). There were 64 experimental trials in total, which lasted for 6 min. In each trial, children saw an array of yellow dots on the left side of the screen and an array of blue dots on the right. They were asked to press a key as quickly as possible on the side of the array containing more dots. The number of dots in each array varied from 5 to 21. In half of the experimental trials, the left array contained more dots, and in half the right. In each trial, the dot arrays were presented for 1,951 ms. To prevent children from basing their judgments on area rather than numerosity, the dot arrays paired in each trial were randomly matched for their total filled area or their individual dot size. The ratio of the dots in each pair fell within one of the following ranges: 1.2–1.37, 1.37–1.57, 1.63–1.87, and 2.57–2.94. Each ratio range appeared 16 times. Before the experimental trials, there were four practice trials in which sound feedback was provided. Children had to repeat the practice trials until they got at least three correct. No feedback was provided during the experimental trials.

##### Number comparison

This task was used to assess children’s symbolic representation of numbers. It was programmed with the software E-Prime (Version 2.0; Schneider et al., 2002). There were 48 experimental trials, which lasted for 5 min. In each trial, children saw two digits ranging from 1 to 9 on the screen. They were asked to press a key on the side corresponding to the numerically larger number as quickly as possible. Half of the trials contained small digits (1–5), while the other half contained large digits (5–9). Among the trials of each digit size, half contained pairs of close digits (numerical distance = 1), whereas the other half contained pairs of digits which were far apart (numerical distance = 3 or 4). Each trial began with a fixation cross appearing on the screen for 1 s. Then a digit pair was displayed until the children responded. Before the experimental trials, children completed four practice trials. They had to repeat the practice trials until they got at least three correct. Both visual feedback (a tick or a cross) and verbal feedback (from the experimenter) were provided during the practice trials. No feedback was provided during the experimental trials.

##### Dot estimation

We used the estimation task developed by Mejias et al. (2012) to assess how well children mapped number words onto approximate representations. The task was programmed with the software E-Prime (Version 2.0; Schneider et al., 2002). There were 16 experimental trials. In each trial, children saw an array of dots in one of the four possible set sizes (i.e., 8, 16, 34, or 64). The children were asked to estimate the number in each array without counting. Each set size was presented four times in random order. Each trial began with a fixation cross appearing on the screen for 1 s. Then an array of dots was displayed on the screen for a maximum duration of 1 s, and the children had to give a verbal estimate of their number. The experimenter wrote down the children’s estimate and the next trial followed. For half of the experimental trials, the total area occupied by the dots was directly proportional to the numerosity; for the other half, the total area occupied by the dots was held constant. This was to prevent children from making estimations based on the total area occupied by the dots. Before the experimental trials, children completed four practice trials showing 15, 50, 20, and 75 dots. At the end of each practice trial, the experimenter told the children the correct number of dots. No feedback was provided during the experimental trials.

## Results

Before analyzing the data, we removed the outliers. For each task, individual scores beyond 2.5 standard deviations from the mean score were excluded from analysis. For the number comparison task, scores below chance level (accuracy lower than 50%) were also excluded. This resulted in excluding 4 children in the numerosity comparison task, 11 children in the number comparison task, and 4 children in the dot estimation task. Hence, the final sample consisted of 85 children for the numerosity comparison task (*n* = 30 for label-and-count, *n* = 30 for label, *n* = 25 for control), 78 children for the number comparison task (*n* = 25 for label-and-count, *n* = 25 for label, *n* = 28 for control), and 85 for the dot estimation task (*n* = 28 for label-and-count, *n* = 29 for label, *n* = 28 for control).

**FIGURE 2:**
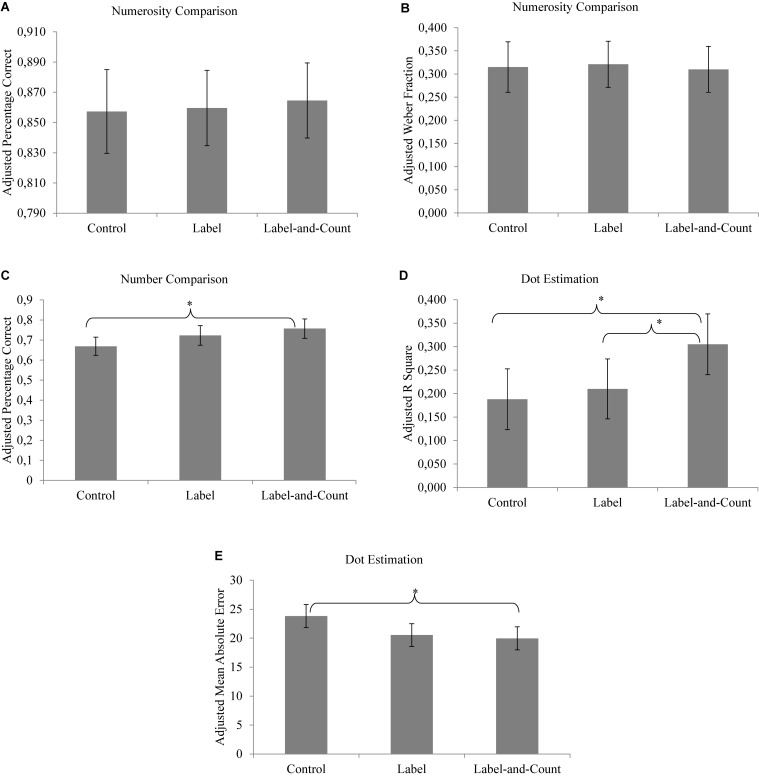
**(A–E)** Adjusted mean posttest scores of children in different reading conditions. Bars present 95% confidence intervals (^∗^*p* < 0.05).

One-way analysis of covariance (ANCOVA) was conducted for each task to compare the posttest scores of the children in the three reading conditions, controlling for their pretest scores. Table 2 shows the mean scores of children in the pretest and posttest. Figures 2A–E compare the adjusted mean posttest scores of the children in the three reading conditions in the posttest. We used Bonferroni adjustment for multiple comparisons.

**TABLE 2:** Mean Scores of children in different reading conditions.

	**Pretest (SD)**				**Posttest (SD)**			
		
	**Numerosity comparison**		**Number comparison**	**Dot estimation**	**Numerosity comparison**		**Number comparison**	**Dot estimation**
						
**Reading condition**	**Percentage correct**	**Weber fraction**	**Percentage correct**	**Mean absolute error**	**Percentage correct**	**Weber fraction**	**Percentage correct**	**Mean absolute error**
Control	0.89 (0.06)	0.28 (0.11)	0.69 (0.18)	20.89 (2.62)	0.87 (0.07)	0.30 (0.11)	0.69 (0.19)	23.43 (7.62)
Label	0.84 (0.09)	0.36 (0.2)	0.63 (0.16)	21.05 (4.67)	0.85 (0.08)	0.34 (0.17)	0.70 (0.17)	20.52 (3.27)
Label-and-Count	0.85 (0.08)	0.34 (0.15)	0.65 (0.17)	20.86 (3.94)	0.86 (0.08)	0.31 (0.13)	0.74 (0.19)	20.10 (4.67)
								

### Numerosity Comparison

We used percentage correct and Weber fraction as indices for the acuity of the approximate number system. Weber fraction determines the increase in percentage correct with increasing number ratios. Unlike percentage correct, the smaller the Weber fraction, the more precise the approximate number system. The Panamath program automatically calculated the Weber fraction for each child (Halberda et al., 2008). There were five children whose Weber fractions in either pretest or posttest were infinity and thus were not included in the subsequent analysis. In the analysis of covariance (see Figures 2A,B), we did not find any effects of reading condition for percentage correct [*F*(2,81) = 0.081, *p* > 0.05] or for Weber fraction [*F*(2,76) = 0.049, *p* > 0.50]. This suggested that children having participated in different reading conditions did not show any difference in their acuity of the approximate number system.

### Number Comparison

We calculated the percentage correct for each child. In the analysis of covariance (see Figure 2C), the main effect of reading condition was significant, *F*(2,74) = 3.48, *p* = 0.036, η_p_^2^ = 0.086. Children in the label-and-count condition were more accurate when comparing numbers than their peers in the control condition (*p* = 0.033), suggesting that counting significantly enhanced children’s symbolic representation of numbers. No other significant difference was found.

### Dot Estimation

If a child mapped the number words well onto the approximate representations, his or her verbal estimates would increase with the numerosities linearly. This can be assessed by calculating the variance (*R*^2^) accounted for by the best-fitting linear function between the actual set sizes and the corresponding estimates by the child (larger explained variance means better fit and thus better mapping). In the analysis of covariance (see Figure 2D), the main effect of reading condition was significant [*F*(2,81) = 3.65, *p* = 0.030, η_p_^2^ = 0.083]. The linear function yielded by the children in the label-and-count condition showed better fit (adjusted *R*^2^ = 31%) than that in the label condition (adjusted *R*^2^ = 21%; *p* = 0.041) or the control condition (adjusted *R*^2^ = 19%; *p* = 0.013), whereas the latter two did not differ (*p* > 0.05). This suggests that children who had counted and labeled the sets showed better symbolic–non-symbolic mapping than their peers who had not done so.

To capture the precision of estimation, we followed Mejias et al. (2012) to calculate the mean absolute error for each child. For example, if a child was shown an array of 34 dots and estimated the quantity as 25, then the absolute error score for this trial would be 9. The mean absolute error measured the average deviations of a child’s estimates from the actual sizes. In the analysis of covariance (see Figure 2E), the main effect of reading condition was significant, *F*(2,81) = 4.33, *p* = 0.016, η_p_^2^ = 0.097. Children in the label-and-count condition estimated more accurately than those in the control condition (*p* = 0.024). Children in the label condition were not significantly more accurate than those in the control condition (*p* = 0.065). Children in the label-and-count condition and label condition yielded comparable estimation accuracy (*p* > 0.05). Taken together, these findings indicate that children who had counted and labeled the sets showed more precise estimation than their peers who had neither counted nor labeled the sets.

## Discussion

Humans possess two representational systems of numbers: the innate approximate number system and the acquired number word system. The two systems are related and mapped onto each other. Yet it remains unclear whether the development of such mapping ability depends on counting skill. To our best knowledge, this study is the first to fill the gap and go beyond correlational studies by using an intervention design. In particular, we ask whether counting the sets of items in the story while reading would lead to better mapping between number words and approximate magnitudes among preschool children.

### Symbolic–Non-symbolic Mappings

We found that children in the label-and-count condition showed better mapping between symbolic and non-symbolic representations after hearing the narrator label the quantities of the items in the story and count them, compared with their peers in the label condition who had heard the narrator label the quantities only, or their peers in the control condition who had neither heard the narrator label the quantities neither nor counted the items. This suggests that counting enhances mappings between number words and approximate representations, a position which is supported by previous studies (Lipton and Spelke, 2005; Sarnecka et al., 2015). We did not find better symbolic–non-symbolic mappings among the children in the label condition than those in the control condition, which suggests that learning the cardinalities of individual number words alone, without making connections with other numbers in the number sequence, may not be sufficient to enhance overall mappings between number words and approximate representations.

These findings may be explained by the way number words are mapped onto approximate representations. Previous studies have suggested two possible mechanisms: associative mapping and structure mapping. In associative mapping, each number word is mapped individually onto its corresponding approximate representation through specific word-magnitude pairings. Such item-specific associations are independent of one another. Moreover, knowledge of the exact cardinal value of each number word is probably necessary. Previous studies suggest that people rely on associative mapping when they link small number words (up to three or four) to non-symbolic magnitudes (Sullivan and Barner, 2013). This explains why adults can produce almost errorless estimates for sets up to four items and their estimates are independent of the experimental contexts (e.g., whether the set of items are presented in a canonical or random pattern; Mandler and Shebo, 1982).

In contrast, in structure mapping, the symbolic and non-symbolic systems are mapped onto each other based on the structural similarities between the two (Gentner and Namy, 2006; Carey, 2009). Since both systems share ordinal structure, people connect them to support estimation. In that sense, individual number word mappings depend on each other. Unlike associative mapping, structure mapping has more to do with the ordinality of numbers than their cardinality. People rely on structure mapping when they link relatively large numbers (beyond four) to non-symbolic magnitudes (Izard and Dehaene, 2008; Sullivan et al., 2011; Sullivan and Barner, 2013). Hence, when estimating large sets, adults adjust their estimates depending on the calibration provided (Izard and Dehaene, 2008). For example, when the adults are explicitly told the number of items contained in a given display, they will estimate the number of items in the rest of the displays accordingly.

In our study, the mappings required in the dot estimation task had to do with relatively large set sizes (beyond four). Hence, structure mapping is probably involved, in which each number is mapped onto the target set according to its ordinality. A number which comes later in the count sequence would be mapped onto a larger set. Learning to count probably helps children build up a better sense of the ordinality of numbers and thus facilitates structure mapping for relatively large numbers. Note that labeling without counting the sets while reading stories simply exposes children to the exact cardinality of the number words. This may help one-on-one associative mappings of small numbers (up to four), but may not be very useful for structure mapping of relatively large numbers.

However, in the study by Barth et al. (2009), children’s mapping ability exhibited little connection to their counting ability as some of them can map number words onto numerosities beyond their counting range. One possible reason is that having a partial sense of the ordinality of large number words is crucial to initiate the structure mappings for these numbers and that having better knowledge of the precise positions of these numbers in the count sequence will lead to better mappings. Children do have some sense of how large numbers relate to each other before they can integrate these numbers into their count sequence precisely (Mix et al., 2014). For example, they may have a sense that 40 is somewhat smaller or earlier in the count sequence than 60 although they cannot count to 60 without error. This partial sense of the ordinality of large numbers probably helps children map large, unfamiliar number words onto numerosities in a somewhat linear manner. As children learn to count to these large numbers and thus have better knowledge of their precise positions in the count sequence, they form a better mapping spectrum between these numbers and their corresponding numerosities.

Counting the items in each set starting from one while reading the stories improves not only the mappings between numbers and set sizes, but also the precision of estimation. Here we found that children in the label-and-count condition produced more accurate estimation than their peers in the control condition. Note that simply labeling the set sizes without counting them (the label condition) was not sufficient to lead to significantly more precise estimation. One possible explanation is that to produce accurate estimates for the relatively large set sizes (beyond four), one may need to have good structure mappings between number words and numerosities and some sense of the exact non-symblic representations of some large numbers (e.g., how a set of 60 items looks in real life). Such a sense may serve as an anchor for associating the rest of the numbers in the count sequence precisely with their corresponding, exact set sizes. In the label-and-count condition, children had learned to count to large numbers, which in turn facilitated better structure mappings, and had practiced pairing up these numbers with their exact set sizes by labeling, which further helped calibrate the structure mappings. Hence, these children turned out to be more accurate in estimation than the children who had neither counted nor labeled the sets when reading the stories.

### Approximate Number System

We found that children in the label-and-count and label conditions were no better than their peers in the control condition when comparing numerosities, suggesting that neither labeling the cardinality nor learning counting could significantly improve the acuity of the approximate number system. This was probably because labeling the cardinality had to do with tagging numbers onto non-symbolic exact representations, whereas verbal counting had to do with ordinality of numbers. Both appeared to work more on the exact number system than the approximate number system. Training directly involving the approximate number system, such as practice comparing two sets of numerosities without counting, has been shown to improve the precision of the system (Wang et al., 2016). One may argue that although practice counting and labeling set sizes appears to target exact number skills rather than approximate number skills, improvement in the former skills may in turn refine the latter (Mussolin et al., 2016). To explore this possibility, future study may consider extending the practices of counting and labeling set sizes over a longer period in order to allow any transfer of gains in the symbolic skills to the non-symbolic skills.

### Symbolic Number System

Children in the label-and-count condition, but not those in the label condition, were more accurate than their peers in the control condition when comparing numbers. This is probably because learning to count helps children better understand the ordinal sequence of numbers. Such knowledge may be useful when comparing two numbers (e.g., knowing that 7 is earlier than 9 in the count sequence helps one decide that 7 is smaller than 9). Since children counted starting from one for each of the five ranges of set sizes in the reading sessions, they had many more chances to practice the sequence for small numbers than large. In our study, the number comparison task involved single-digit numbers only. Future study may include two-digit numbers to fully reveal the benefits of learning counting to number comparison.

### Practical Implications

We used story reading because it is a typical parent-child activity across cultures and many storybooks show pictures of items in varying quantities. Consistent with previous studies (Blevins-Knabe and Musun-Miller, 1996; Young-Loveridge, 2004; Levine et al., 2010; Gunderson and Levine, 2011; Mix et al., 2012), this study has shown that informal everyday exposure might offer valuable input to children’s numeracy development. Based on the present findings, parents and early educators are encouraged to count with children the set of items in story books while reading to the children to help them map the number words onto their preexisting approximate representations. It is not a common practice for parents to count the items in the story books when reading to children (Mix et al., 2012). Hence, our findings challenge the traditional way of story reading and offer new insights into making use of it as a simple, inexpensive way to enhance young children’s symbolic–non-symbolic mappings in daily life. Future research may explore if the same rapid learning in the label-and-count condition applies in languages where the mapping of number words to quantity is more or less transparent than Chinese. Moreover, it is worthwhile to examine whether children struggling with the number word system (e.g., those with language difficulties) will learn best in the label-and-count condition, or if the exposure to written number digits will play a more important role in these children.

## Conclusion

Children make sense of the number word system by mapping numerals onto their corresponding, preexisting approximate representations. Using the case of story reading, this study has shown that counting the items in story books while reading to children enhanced their mappings between number words and approximate representations. This is consistent with previous findings that suggest the importance of verbal counting in forming the mapping spectrum between numbers and numerosities. This entails implications for parents and early educators on how to enhance young children’s symbolic–non-symbolic mappings in daily life.

## Data Availability Statement

The datasets generated for this study are available on request to the corresponding author.

## Ethics Statement

The studies involving human participants were reviewed and approved by the Human Research Ethics Committee, The University of Hong Kong. Written informed consent to participate in this study was provided by the participants’ legal guardian/next of kin.

## Author Contributions

The author contributed conception and design of the study, organized the database, performed the statistical analysis, and wrote the manuscript.

## Conflict of Interest

The author declares that the research was conducted in the absence of any commercial or financial relationships that could be construed as a potential conflict of interest.
